# Flavonoids, Inflammation and Immune System

**DOI:** 10.3390/nu8100659

**Published:** 2016-10-21

**Authors:** Francisco J. Pérez-Cano, Margarida Castell

**Affiliations:** 1 Departament de Bioquímica i Fisiologia, Facultat de Farmàcia i Ciències de l’Alimentació, Universitat de Barcelona (UB), Av. Joan XXIII 27-31, 08028 Barcelona, Spain; franciscoperez@ub.edu; 2 Institut de Recerca en Nutrició i Seguretat Alimentària (INSA), C/Prat de la Riba 171, Santa Coloma de Gramenet, 08921 Barcelona, Spain

**Keywords:** antioxidant, atherosclerosis, autoimmunity, immune response, inflammatory response, cardiovascular disease, cytokine, intestinal inflammation, lymphocyte functionality, macrophage activity

Flavonoids, including around 6000 phenolic compounds, are products of the secondary metabolism of plants which can be a part of one’s diet via the consumption of many edible plants. Flavonoids can be classified into flavonols (such as quercetin, kaempferol, isoquercetin, etc., found in onions, apples, berries, kale, leeks, broccoli, blueberries, red wine and tea), flavones (such as glycosides of luteolin, chrysin and apigenin, commonly found in fruit skins, parsley and celery), isoflavones (such as genistein, daidzein and glycitein present in leguminous plants, mainly soy and soy products), flavanones (such as naringenin, eriodictyol and hesperidin exclusive of citrus fruits), flavanols (such as epicatechin, catechin, gallocatechin, epigallocatechin, epigallocatechin gallate and also polymeric forms or condensed tannins as found in cocoa and tea), and anthocyanidins (such as pelargonidin, cyanidin and malvidin, found in red wine and berry fruits). Chemically, flavonoids have a polyphenolic structure that confers antioxidant activities on them. The antioxidant properties of flavonoids have been recognized since for more than 40 years ago [1] and, in that time (1976–2016) nearly 23,000 publications have appeared (more than 20,000 research articles and 2600 reviews) according to research in the Scopus database (searching ‘flavonoid and antioxidant’). However, flavonoid biological activities go beyond antioxidant properties, although some of them are related to these abilities. Some particular kinds of flavonoids have shown protective effects against cancer [2,3], cardiovascular diseases [4,5,6], gastrointestinal alterations [7] and nervous system-related syndromes, such as depression [8], epilepsy [9,10], Alzheimer’s disease [11] and neurodegenerative disease [12], among other pathologic conditions.

The anti-inflammatory actions of particular flavonoids have also appeared in recent years. The evidence in the literature is based on in vitro and in vivo studies as well as on clinical studies. In an inflammation context, some of the flavonoids’ action mechanisms, such as acting as antioxidants, modulating gene expression (i.e., cytokines, adhesion molecules) or enzyme activities, have been described. However, the role of flavonoids in the specific arm of the immune response still remains quite unexplored. The Special Issue of *Nutrients* entitled, ‘Flavonoids, Inflammation and Immune System’ aimed to encourage researchers to update the knowledge of these compounds from these two, closely related, biological aspects: inflammatory response, mainly conducted by macrophages and neutrophils as an expression of the innate immune system activation; and the immune system concerning, above all, acquired immunity. As a result, this Special Issue contains examples of the interest in these areas worldwide. The articles included here demonstrate the tendency for researchers to explore flavonoids, either as a pure compound or included in food and their impact on cells, animals or humans in reference to inflammatory or immune response. This issue provides an opportunity to update current knowledge about some examples of the six subclasses of the flavonoid compounds and their effects—even at molecular level—on immunity and inflammation, but also in inflammatory-mediated diseases such as insulin-resistance obesity, cardiovascular disease and even in cancer. The Special Issue contains six review papers [13,14,15,16,17,18] and eight research papers [19,20,21,22,23,24,25,26] on the current knowledge in the field. Contributions are from countries in Africa (South Africa), America (Brazil), Asia (China, Korea, Japan and Taiwan) and Europe (Germany, Spain and Switzerland).

The review by Vezza et al. [13] focuses on the effect of flavonoids on inflammatory bowel disease, going in depth into the proposed action mechanisms. These authors report in vitro and in vivo studies about the effect of anthocyanins, chalcones, flavanones, flavones, flavonols, flavanols and isoflavones on inflammatory bowel disease, and they also disclose the lack of clinical trials to confirm the actual role of flavonoids in this inflammatory disease [13]. The review by Goya et al. [14] also includes some of the studies of an important source of flavanols—cocoa—on animal models of colon inflammation. On the other hand, quercetin, a flavonol with long-recognized unique biological properties, is also the focus of a review article by Li et al. [17], who provide a review of the literature about the effects and mechanisms of quercetin on inflammation and immune function in in vitro, in vivo and in clinical studies. Overall, the majority of the literature revised supports the benefits of prolonged supplementation with quercetin. As an example of the varieties of mechanisms of quercetin’s action, a research article in the issue looks in depth at the ability of this flavonol to modulate the sealing of epithelial cells through tight junction gene expression regulation by microRNAs [25]. Another flavonol with well-known biological activities is myricetin. Semwal et al. [18] provide an extensive review of the studies demonstrating, among others, its anti-oxidant, anti-photoaging, anti-cancer, anti-platelet aggregation, anti-hypertensive, anti-inflammatory and immunomodulatory and anti-allergic activities and applications. They conclude that, although more toxicity studies should be developed, myricetin may constitute a new agent for these situations in the near future.

The Special Issue contains only one in vitro study showing that one flavonol—kaempherol—is able to partially inhibit stress-induced inflammation and hepatic insulin resistance in HepG2 cells [23]. On the other hand, although it is well established that the principal pharmacological action of phlorizin, a chalcone, is to produce renal glycosuria and to block intestinal glucose absorption through inhibition of the renal and mucosal sodium–glucose symporters, one article included in this issue highlights other complementary effects using an in vivo approach in obese mice, such as its action on hepatic steatosis, inflammation and fibrosis [26].

Cocoa, as a food relatively rich in flavonoids, is the focus of several articles in this issue. There are two reviews that compile studies demonstrating the anti-inflammatory action of cocoa consumption. Goya et al. [14] focus on inflammation as a pathogenic mechanism involved in cardiovascular disease and they compile in vitro studies as well as approaches in experimental animals showing its particular effect on inflammation markers. Moreover, they comment on the most recent publications on the effects of cocoa on anti-inflammatory markers in human cohorts. In this context, the critical review by Ellinger et al. [15], who include 33 randomized, controlled trials reporting the effects of cocoa consumption on inflammatory biomarkers, is important. Nevertheless, these authors conclude that the evidence for the anti-inflammatory effects of cocoa is currently scarce, although cocoa consumption may prevent or even reduce vascular inflammation. Staying with the effects of cocoa and chocolate, this Special Issue also includes a research article [21] focused on a cross-over, placebo-controlled, double-blind, randomized clinical trial conducted in 92 patients infected with human immunodeficiency virus (HIV)—a population with a high cardiovascular risk. This study also includes another food rich in polyphenols—yerba mate. The conclusion of this first clinical study to evaluate the effect of those flavonoids on the inflammatory profile of such patients undergoing antiretroviral therapy, indicates that 65 g dark chocolate is able to increase high-density lipoprotein cholesterol (HDL-c) concentrations [21]. A different effect of cocoa is shown by Camps-Bossacoma et al. [22], who reported the tolerogenic effects of cocoa on a rat model of oral sensitization. This study, focused on acquired immunity, reveals the changes produced by cocoa intake on mesenteric lymph nodes, which eventually prevent oral immunization to a food allergen. Gut-associated lymphoid tissue is also evaluated in the study by Martín-Peláez et al. [20], who included a clinical trial with olive oil phenolic compounds. This randomized, controlled, double-blind, cross-over human trial demonstrates that the consumption of a virgin olive oil containing 500 mg/kg olive oil phenolic compounds increases the proportion of intestinal bacterial coated to IgA [20]. Another clinical study (open, prospective, randomized, cross-over, controlled feeding trial) includes the effect of tomato and olive oil on cardiovascular disease risk factors [19]. This article reports that a single tomato intake, especially tomato sauce enriched with refined olive oil, decreases plasma total cholesterol, triglycerides and several cellular and plasma inflammatory biomarkers, whereas it increases HDL-c concentration.

On the other hand, although some extracts are not sufficiently characterized in order to precisely know the flavonoid pattern present, they have been shown to be effective on several inflammatory disorders. This is the case for the extract of *Zingiber zerumber*, very rich in flavonoids and with significant protective effects on retinal inflammation [24].

Finally, it has to be taken into account that, despite the increasing reports about the benefits of flavonoids on inflammation, knowledge about their potential health risks is also provided. In this issue, Yu et al. [16] examine the anti-inflammatory benefits of isoflavones, but also review the current evidence of their negative health effects.

We hope that the articles contained within this issue, but also the references they include and comment on, are of interest to researchers, clinicians, dieticians and the rest of professionals or non-professionals involved in the passionate world of the interaction between diet—particularly those with food rich in flavonoids—and health. Besides the update in the knowledge about the particular actions of flavonoids on immunity and inflammation, we also encourage further clinical trials and experimental research to characterize intracellular action mechanisms, to establish the amount of flavonoids needed to achieve such effects and also to establish the precise action of each particular flavonoid or extract containing flavonoids.
